# PhyloFunDB: A Pipeline to Create and Update Functional Gene Taxonomic Databases

**DOI:** 10.3390/microorganisms10061093

**Published:** 2022-05-25

**Authors:** Ohana Y. A. Costa, Mattias de Hollander, Eiko E. Kuramae, Paul L. E. Bodelier

**Affiliations:** 1 Department of Microbial Ecology, Netherlands Institute of Ecology (NIOO-KNAW), 6708 PB Wageningen, The Netherlands; o.costa@nioo.knaw.nl (O.Y.A.C.); m.dehollander@nioo.knaw.nl (M.d.H.); e.kuramae@nioo.knaw.nl (E.E.K.); 2 Department of Terrestrial Ecology Bioinformatics Unit, Netherlands Institute of Ecology (NIOO-KNAW), 6708 PB Wageningen, The Netherlands; 3 Ecology and Biodiversity Group, Department of Biology, Institute of Environmental Biology, Utrecht University, Padualaan 8, 3584 CH Utrecht, The Netherlands

**Keywords:** functional genes, taxonomy, pipeline

## Abstract

The increase in sequencing capacity has amplified the number of taxonomically unclassified sequences in most databases. The classification of such sequences demands phylogenetic tree construction and comparison to currently classified sequences, a process that demands the processing of large amounts of data and use of several different software. Here, we present PhyloFunDB, a pipeline for extracting, processing, and inferring phylogenetic trees from specific functional genes. The goal of our work is to decrease processing time and facilitate the grouping of sequences that can be used for improved taxonomic classification of functional gene datasets.

## 1. Introduction

Several studies employ the amplification of functional genes for taxonomic profiling of specific microbial communities, such as genes involved in nitrogen (*nirK*, *nosZ*, *amoA*) or carbon (*pmoA*, *mcrA*) cycling. However, the taxonomic classification relies on databases, which requires representatives of as many taxonomic groups as possible in order to obtain the most reliable taxonomic assignments [1]. Developments in high-throughput sequencing have increased the number of unclassified sequences in databases such as Genbank, as most sequences are derived from cultivation-independent studies [2,3,4]. Grouping sequences by similarity, analyzing their clusters in phylogenetic trees, and comparing the position of unknown sequences to known clades is a recognized approach for improving the taxonomy of currently unclassified sequences [4]. This process, however, demands the download and processing of sometimes large amounts of data using several different software, a procedure that we aim to facilitate and integrate with PhyloFunDB.

Here, we present PhyloFunDB, a pipeline for extracting specific genes of choice, processing sequences, and inferring phylogenetic trees that can be used to assign or confirm microbial taxonomic groups. Such groups can be employed for an improved taxonomic classification of functional gene datasets. Additionally, we provide a second configuration option to update the databases and trees generated by the pipeline, where the newest sequences can be extracted, processed, and added to the databases and phylogenetic trees that need updates.

In order to demonstrate the functionality of the pipeline, we chose the *mcrA* gene to perform the procedure. The *mcrA* gene produces the alpha subunit of the methyl coenzyme M reductase complex, which is fundamental in the archaeal methane metabolism [5,6]. Methanogenic communities play an important role in the global greenhouse gas budget, as they produce methane under anoxic conditions and have been observed in wetlands, sediments, permafrost areas, rice pad digesters, geothermal springs, and hydrothermal vents [3,7,8]. The workflow of our pipeline is inspired by the methods Alves et al. [9] used to perform a global phylogenetic analysis of archaeal *amoA* genes. The pipeline is implemented in Snakemake [10].

## 2. Materials and Methods

PhyloFunDB is a pipeline that expedites the production of functional gene databases for taxonomic assignment. Based on the workflow of Alves et al. [9], it integrates NCBI Entrez API [11], Mothur [12], MAFFT [13], FrameBot [14], IQ-TREE [15], and RAxML [16], all the tools packaged in conda (https://docs.conda.io/en/latest/, accessed on 17 May 2022) environments to facilitate installation. Alves et al. [9] downloaded more than 30.000 sequences and processed and inferred phylogenetic trees in order to reconstruct a highly resolved phylogeny of the archaeal *amoA* gene. Inspired by their workflow, we compiled software to automate the processes of primary sequence selection and filtering, trimming, and phylogenetic tree inference, speeding up the process and allowing the reproducible generation of databases for several different genes at the same time, with one command line only.

**Figure 1:**
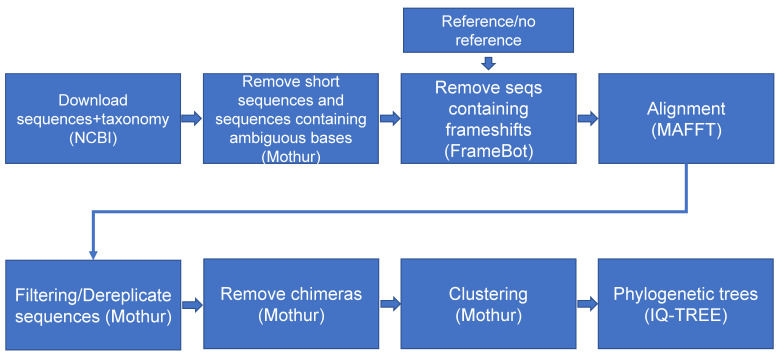
Workflow of the database-producing PhyloFunDb pipeline. Sequence download, processing, filtering, clustering, and tree inference.

**Figure 2:**
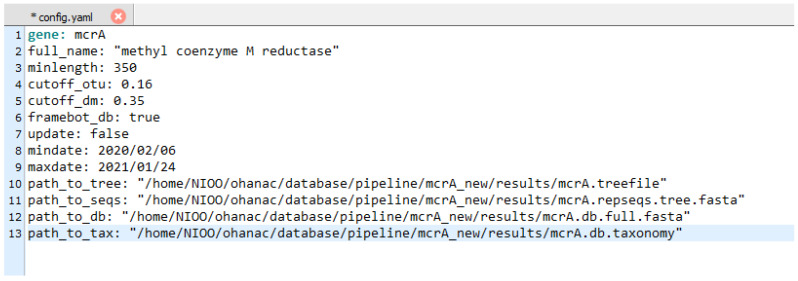
Configuration file with parameters defined by the user. Configuration file for the database producing pipeline, containing parameters for query search, minimum sequence size, OTU and distance matrix cutoff, and FrameBot database status. When the update parameter is set to true, the user also has to input the date range for the download of the new sequences and paths to the files of the database to be updated.

PhyloFunDB is based on the analysis of nucleotide sequences and not on amino acid sequences, since our aim is to target taxonomy, and nucleotide sequences potentially provide higher phylogenetic resolution at the most recent phylogenetic scales [4]. The workflow illustrated in Figure 1 starts with a NCBI query search, with parameters defined by the user. In the configuration file *config.yaml* (Figure 2), the user provides the gene of interest, as well as the full name of the function/gene, needed for NCBI query search and gene region extraction by NCBI Entrez API. It looks for the gene of interest in NCBI database [17], obtains the records in xml format, extracts information of the start and the end of the gene, sorts and removes duplicated accession numbers, and lastly, recovers the fasta format DNA sequences containing the accession number and gene of interest. The NCBI taxonomy string associated with each one of the downloaded accession numbers is also retrieved, in order to facilitate further manual curation of the taxonomy. Next, sequences containing the word “UNVERIFIED” are removed, and Mothur [12] trims the sequences to the minimum size defined by the user. FrameBot [14] performs filtering and frameshift correction of the sequences, based on a curated protein database, which can be downloaded from FunGene (http://fungene.cme.msu.edu/, accessed on 17 May 2022), or skipped, in case there is no database available by setting the parameter *framebot_db* in the config file to *false*. Following this, the remaining sequences are aligned by MAFFT [13] using the auto option, and thus the software automatically selects an appropriate alignment strategy. This option can be adjusted by the user in the *Snakefile*. Next, the alignment is filtered and screened, and redundant sequences are removed by Mothur. In addition, Mothur, using the most abundant sequences in the dataset as reference, performs a chimera (artifacts generated by PCR) removal. Then, the alignment is screened and filtered again, and the sequences are clustered in OTUs (operational taxonomic units), based on a distance matrix calculation by Mothur, using the average clustering method that can be modified by the user in the *Snakefile*. Usually, the default parameter for the distance matrix (*cutoff_dm*) is enough, but if the OTU cutoff is higher than 0.12, then the distance matrix cutoff should be also increased, otherwise the program will not perform the clustering at the chosen cutoff. For *mcrA* analysis, we chose 0.16 as cutoff for OTU clustering [18], 0.35 as cutoff for the distance matrix, and a minimum of 350 base pair sequences. The OTU grouping, instead of ASV (amplicon sequencing variant) analysis, is applied in order to decrease the complexity of the dataset and facilitate downstream manual curation, as the datasets may contain more than 30,000 single sequences. However, if the user desires to include all sequences in the tree, the *cutoff_otu* parameter can be set to 0.01. Next, the OTU representatives are extracted and used by IQ-TREE [15] for tree inference, using ModelFinder [19] for best model selection and 1000 ultrafast bootstraps [20] for branch support values. Output files from PhyloFunDB include the .*treefile* with the OTU representatives, a .*fasta* file containing the complete set of sequences, and the associated *.txt* taxonomy file, which can be used for the manual curation of the database.

**Figure 3:**
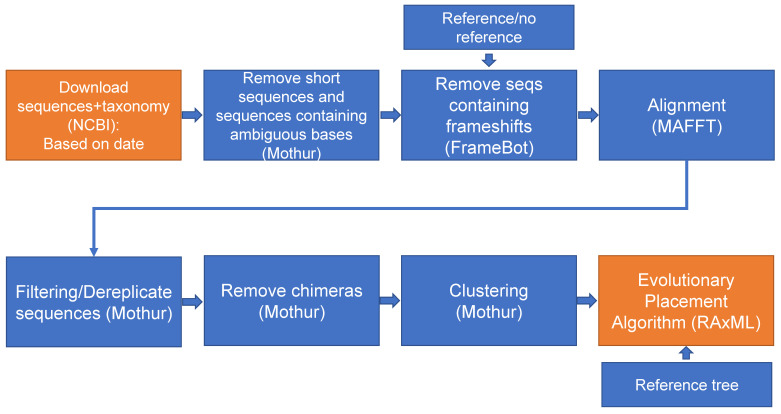
Workflow of the database-updating option of the PhyloFunDb pipeline. New sequences are downloaded, processed, filtered, clustered, and added to the tree and to the original database files. The steps that are different from the database-producing option are shown in orange.

Moreover, after building a specific gene database, updating with new sequences is also possible. A second configuration option is available (Figure 3), for downloading and processing the newest sequences uploaded to the NCBI database. In the *config.yaml* file (Figure 2), the user can set the parameter *update* to *true* and add a date range for downloading new sequences, which go through the same downstream processing as in the first workflow. At the end, however, only the new OTUs are added to the reference tree, and the fasta sequences are appended to the old fasta file. The new OTUs are placed in the reference tree by using the evolutionary placement algorithm (EPA) [21] from RAxML. In the *config.yaml* file, the user also provides the paths to the reference tree and database archives where the new sequences should be appended (*path_to_tree*, *path_to_seqs*, *path_to_db* and *path_to_tax* parameters).

## 3. Results and Discussion

It took approximately 40 h to retrieve and process 31,202 sequences, and to generate the final archives using 8 CPU threads on a shared multicore server with ample compute power (80 Intel Xeon Gold 6230 CPU threads, 75 GB of RAM) running Ubuntu 20.04 LTS. Retrieving sequences from NCBI and frameshift correction by FrameBot [14] were the longest steps (approximately 24 and 14 h, respectively). Following this, we performed the manual checking of *mcrA* OTUs representatives and groups. After obtaining the database files, the manual curation is necessary to check the taxonomy/clustering of the sequences in the inferred tree and improve the classification of the unassigned/unclassified sequences. In addition to the appended taxonomy, it is also possible to download metadata from all sequences using the NCBI Entrez API and add that information to the created database. For downloading metadata associated with our *mcrA* gene sequences, we used the following command line: “esearch -db nucleotide -query “mcrA[gene]”|efetch -format gpc|xtract -insd source organism mol_type strain country isolation_source|sort|uniq >metadata_mcrA.txt”.

**Figure 4:**
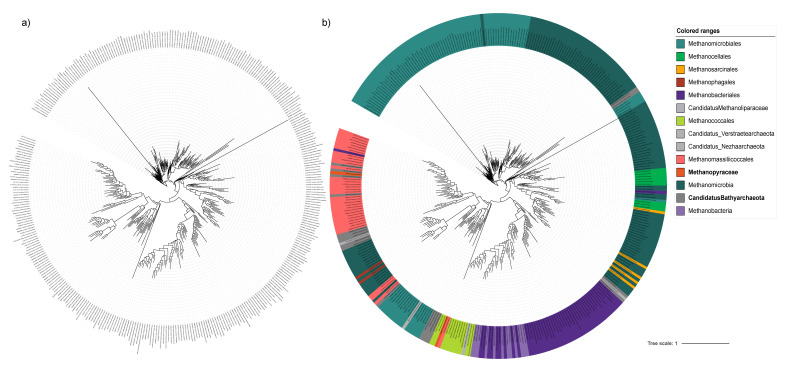
Phylogenetic trees inferred by the workflow of PhyloFunDb pipeline used to create a *mcrA* gene database. (**a**) Inferred tree created as output of PhyloFunDb; (**b**) after manual curation of the sequence classification, several sequences coming from environmental studies had their classification improved.

After downloading the metadata, we checked whether there were cultivated representatives within the OTU groups, as the OTU representative sequence chosen by Mothur is not always a cultivated/known organism. It can be checked in the file “interm/{gene}.aligned.good.filter.unique.pick.good.filter.an.{cutoff_otu}.rep.names”. Next, we used the taxonomy of the known sequences to improve the taxonomy of unknown sequences. Unclassified clusters were identified in the inferred tree based on the closest classified sequences (Figure 4). Many of the sequences were identified as order-like or family-like classifications, due to a lack of a close classified representative. In general, the analysis will also depend on how many sequences from the database are well classified, which varies depending on the gene. Using the metadata downloaded from NCBI, it is also possible to add the environmental origin of the sequences to their taxonomy string. After checking and refining or correcting the taxonomy strings of the OTU representatives, we expanded the taxonomy to all the sequences in the OTU groups using the *expand_taxonomy.R* script, available with the pipeline.

After the manual curation, we obtained a database with 31,202 sequences, containing 1112 sequences classified at least to family level, which allowed the improvement the classification of the other 30,090 sequences. The tree possessed 142 representatives classified at genus level, which helped to improve the grouping and classification of other 219 OTUs. This analysis helped improving the classification of several unclassified sequences, which can be used as a support in amplificon studies to give a better overview of the taxonomy of *mcrA* datasets. However, supplementary analysis, such as protein sequence trees, and more information about the origin of the sequences should be included in order to properly resolve *mcrA* phylogeny.

It is worth noting that we are applying a distance clustering method, which groups taxa by perceived similarity among sequences, instead of evolutionary descent, which can, therefore, result in classification errors. This approach was chosen to decrease the complexity of the datasets, which, otherwise, would result in the individual analyses of thousands of sequences. Nonetheless, the user can avoid the clustering step, including every single sequence in the final tree; however, the processing time will depend on the final number of sequences and bootstrap test may take weeks or even months. Running the pipeline does not require a large number of threads or RAM memory, for instance, running mcrA gene used approximately 2.5 gb. The longer steps are due to the availability of NCBI servers and FrameBot software v 1.2.0 (East Lansing, MI, USA) [14]. FrameBot is a user-friendly java-based tool that, even though useful, has not been improved in recent years. An improvement in the performance of this tool would require the involvement of its original developers. Nonetheless, the speed of the pipeline will depend on the number of sequences employed. The tools we chose do not use considerable resources and were suitable for our purposes; however, comparisons with other clustering and alignment software could be performed, decreasing the processing time even more and increasing group placement accuracy in the phylogenetic trees. Among interesting options are the clustering software CD-HIT and Swarm [22], aligners PRANK [23] and Clustal Omega [24], and HMM-FRAME [25] for frameshift detection and correction.

To our knowledge, there are no pipelines for gene database creation that download sequences and produces phylogenetic trees. The most similar pipeline is SATé [26], which performs sequence alignment and tree estimation, and performance comparisons could be performed as future steps towards improvement of the pipeline. Further enhancements could include capacity to handle several genes at the same time, since the current best way to produce databases for more than one gene is to run the pipeline for the different genes in parallel. In addition, tests with different parameters and software for more accurate alignments, clustering, and tree generation could be executed.

Overall, PhyloFunDB allowed automated retrieval and processing of a considerable number of sequences, which were further used for the evaluation and improvement of *mcrA* gene taxonomy. Further analysis can be performed by the user to produce deeper analyses, including the metadata made available together with the sequences downloaded. In addition, PhyloFunDB can be used in parallel for the processing of several different genes at the same time, speeding up the production of a collection of gene databases.

## Data Availability

The pipeline presented in this study is available at [27].
